# Factors influencing male involvement in maternal and child health care from the health center of Xai-Xai city, Mozambique: a cross-sectional qualitative study

**DOI:** 10.3389/frph.2026.1746689

**Published:** 2026-04-24

**Authors:** Maudy Simão Muchanga, Osvaldo Bernardo Muchanga, Emidio Filipe Dique, Virgílio Simione Cau, Agrácio Ilidio Cumbe, Mariza Manguele, Hilário Antonio Massango, Alfredo Jaime Muchanga, Soares Miguel Michal, Inocêncio Jaime Muchanga, Avelino Nelson Mazuze, Izaidino Jaime Muchanga

**Affiliations:** 1 Associação Sekeleca U Phatima (ASUPHA), Inhambane; 2 Instituto Superior de Gestão e Empreendedorismo Gwaza Muthini, Xai-Xai; 3 Laboratório Provincial de Higiene, Água e Alimentos de Gaza; 4 Escola Secundária de Mahamba, Zavala; 5 Universidade São Tomás de Moçambique, Macia; 6 Universidade São Tomás de Moçambique, Xai-Xai; 7 Instituto Superior Politécnico de Gaza; 8 Hospital Distrital de Jangamo, Inhambane; 9 Universidade Católica de Moçambique, Pemba; 10 Hospital Distrital de Mapai; 11 Universidade Lúrio-Faculdade de Ciências de Saúde, Nampula

**Keywords:** child health, male involvement, maternal health, prenatal care, sociocultural barriers, Mozambique

## Abstract

**Introduction:**

Male participation in maternal and child healthcare (MCH) is essential for improving health outcomes for women and children. Despite evidence that male involvement enhances adherence to medical recommendations and reduces maternal and infant mortality, pregnancy and childbirth are traditionally viewed as women's responsibilities in many settings in Mozambique. This study aimed to explore women's perceptions of the factors influencing the low participation of male partners in MCH at the Xai-Xai City Health Center, Mozambique.

**Material and methods:**

A qualitative cross-sectional study was conducted between June and August 2022. Purposive sampling was used to recruit 15 women aged 18–35 years attending antenatal care or well-child consultations without their partners. Data were collected through semi-structured interviews and analyzed using inductive content analysis with MAXQDA software. Two researchers independently coded the transcripts and resolved discrepancies through discussion until consensus was reached. Data saturation was considered achieved when no new themes emerged from successive interviews.

**Results:**

The study included 15 women, most aged 18–24 years (60%) and residing in urban areas (60%). Participants identified multiple barriers to male participation in maternal and child healthcare. Sociocultural norms positioned pregnancy and childcare as women's responsibilities, contributing to male absence. Economic constraints, including work obligations and transport costs, further limited participation. Fear of HIV testing, associated with stigma and potential relationship conflict, emerged as an additional deterrent. Health system barriers, such as long waiting times, limited privacy, and clinic hours incompatible with men's work schedules, were also reported. Despite these challenges, women generally expressed satisfaction with the quality of care, although concerns about confidentiality persisted.

**Conclusions:**

Male participation in maternal and child healthcare in Xai-Xai City remains constrained by interconnected sociocultural, economic, HIV-related, and health system barriers. Addressing these challenges requires gender-transformative and health system responsive strategies, including male-friendly services, improved confidentiality, and community-based interventions to promote shared responsibility for maternal and child health. These findings provide a localized evidence base for the Ministry of Health to strengthen family-centered care in high-HIV-prevalence to improve health policies in Gaza Province.

## Introduction

Male involvement in maternal and child health (MCH) has increasingly been recognized as an important determinant of positive maternal and neonatal outcomes (1). In the context of reproductive and family health, male involvement generally refers to men's active participation in activities related to pregnancy, antenatal care (ANC), support during childbirth, and engagement in childcare and health decision-making (2). Evidence shows that when male partners participate in these activities, women are more likely to attend antenatal services, adhere to medical recommendations, prepare adequately for childbirth, and seek timely healthcare for themselves and their children (3, 4).

Understanding male involvement in MCH also requires considering broader conceptual perspectives, gender norms theory and masculinity frameworks suggest that socially constructed expectations about male and female roles influence health behaviors and decision-making within households (5, 6). In many societies, reproductive health is culturally defined as a woman's domain, while men are expected to assume roles primarily as financial providers rather than direct caregivers (7). In addition, health-seeking behavior models highlight that decisions about using health services are shaped by cultural norms, perceived benefits, structural barriers, and interpersonal dynamics within families (8). These perspectives provide an important analytical lens for understanding why men may remain absent from maternal and child healthcare services even when such participation is encouraged by health systems.

Across Sub-Saharan Africa, sociocultural norms frequently position pregnancy and childbirth as exclusively women's responsibilities, as a result, it remains uncommon for men to accompany their partners to antenatal care visits or to participate in childbirth-related activities (9).

Even in settings where policies encourage male participation, structural and cultural barriers continue to limit their involvement. These barriers include rigid gender roles, fear of stigma, work-related constraints, and limited awareness of the importance of male engagement in reproductive health (10, 11).

Low male involvement in MCH services has been widely reported across the African continent. Studies conducted in several countries report that male partner attendance at antenatal services remains limited, in Cameroon, despite interventions encouraging men to accompany their partners to obstetric and PMTCT services, only about 18% of male partners participated in such care (12–14). Similarly, a systematic review of male participation in antenatal HIV testing and PMTCT programs across Africa found that attendance ranged from approximately 5%–33%, with particularly low rates in Uganda (less than 5%) and around 15% in Kenya and Tanzania (15–19). A community-based study in Northwest Ethiopia also reported that only 21.2% of male partners were involved in PMTCT activities (14). These findings highlight the persistence of low male engagement despite growing policy attention to this issue.

Although male participation is important for maternal and child health broadly, the topic has also received particular attention in the context of Human immunodeficiency virus (HIV) prevention of mother-to-child transmission (PMTCT). Male partner support has been associated with increased uptake of HIV testing, improved adherence to antiretroviral therapy, and better retention in care among pregnant women (20). However, while HIV related services have often served as entry points for promoting male engagement, male involvement remains relevant to the wider continuum of maternal and child healthcare, including antenatal visits, delivery preparation, and postnatal support (3, 21).

In Mozambique, male involvement in maternal and child healthcare remains limited despite national and international efforts to promote family-centered health services (22). Cultural norms, traditional gender roles, and limited awareness about the benefits of male participation continue to restrict men's engagement in reproductive health services (12, 14). In many communities, pregnancy and childbirth are still perceived primarily as women's responsibilities, leading to minimal male presence in antenatal clinics or maternity wards (22). This absence may contribute to delayed health-seeking behavior, reduced shared decision-making within households, and limited emotional and practical support for women during pregnancy and early childcare (23).

Several studies conducted in Mozambique have explored male partner involvement in MCH services in provinces such as Maputo (14), Zambézia (3, 24), Nampula (11), and Sofala (11). However, despite evidence from other studies conducted in Mozambique, information from Gaza Province particularly from the Xai-Xai City remains scarce. This study is the first to be conducted in the city on this topic and may provide important evidence to inform health policies aimed at promoting male engagement in accompanying their partners to healthcare services. The Xai-Xai City has one of the highest HIV prevalence rates in the country. Despite this epidemiological context, little research has reported male participation in maternal and child healthcare services in this setting.

In this study, male involvement in maternal and child healthcare is understood as the participation of male partners in activities related to antenatal care attendance, support during pregnancy and childbirth, and engagement in decisions and practices related to maternal and child wellbeing. This study aimed to explore women's perceptions of the barriers to male partner involvement in maternal and child healthcare at the Xai-Xai City Health Center in Gaza Province, Mozambique. Understanding women's perspectives of the limited participation of male partners in maternal and child health services and seeks to inform strategies to enhance male engagement.

## Material and methods

### Study area

The study was conducted between June and August 2022 at the Xai-Xai City Health Center, located in Bairro A, Xai-Xai District, Gaza Province, Mozambique. The health center is classified as a Level I healthcare facility within the national health system and serves as the district referral center for primary healthcare services. The study setting provides outpatient maternal and child health services to the local population.

### Study design and sampling methods

A cross-sectional qualitative study design was used to explore women's perceptions and experiences regarding the non-participation of male partners in maternal and child healthcare. In this context, the cross-sectional approach refers to the collection of qualitative data at a single point in time during the study period, capturing participants' experiences and perceptions as they existed during their health facility visit.

This design allowed the researchers to obtain an in-depth understanding of the phenomenon within the specific social and healthcare context in which the participants were receiving services. The sample size is guided by the principle of data saturation rather than statistical representativeness. Data saturation was reached when additional interviews no longer generated new themes or meaningful insights relevant to the research question. During the analysis process, the researchers observed repetition of key ideas and patterns across participants' narratives, indicating that the collected data were sufficient to capture the main dimensions of women's experiences related to male partner non-participation in maternal and child healthcare.

A purposive sampling strategy was used to recruit women who could provide relevant information about the research topic, specifically women attending maternal and child health services without their partners. Within this purposive framework, participants were recruited consecutively during the study period as eligible women attended the health facility and agreed to participate. This approach allowed the researchers to ensure that participants met the inclusion criteria while also facilitating practical recruitment within the clinical setting.

Women aged 18–35 years were included because this age group represents most reproductive-age women accessing maternal and child health services in the study setting. Additionally, including adults ensured that participants could independently provide informed consent. Adolescents under 18 years were excluded due to additional ethical requirements for research involving minors. Women older than 35 years were not excluded intentionally but were not encountered during the recruitment period among those meeting the study criteria and attending the selected services.

### Data collection and analysis

#### Data collection

Data were collected at the study site in accordance with the approved research protocol. Prior to each interview, the interviewer explained the objectives of the study to all participants, emphasizing ethical principles such as confidentiality, anonymity, and voluntary participation. Participants were informed that their identities would not be linked to the information provided and that the data would be used exclusively for research purposes. They were also reminded of their right to withdraw from the interview or the study at any time without any consequences.

The study was conducted in the Adolescent and Youth Health Services (SAAJ), Antenatal Care (ANC), Well-Child Consultations (WCC), and At-Risk Child Consultations (ARCC). These services play a key role in promoting male involvement in maternal and child healthcare, particularly by encouraging adherence to Antiretroviral Therapy (ART), family planning, and follow-up of pediatric consultations. This setting was intentionally selected due to the observed low participation of male partners, making it representative of the broader context of limited male involvement in maternal and child health services.

Data were collected through semi-structured individual interviews (Supplementary Material), consistent with the qualitative nature of the study. This method allowed for an in-depth exploration of participants' perceptions and experiences related to low male participation in maternal and child healthcare. The interviews followed a pre-established interview guide composed of open-ended questions, enabling both consistency across interviews and flexibility to probe emerging themes. The questions focused on understanding women's feelings, perceptions, and experiences regarding the absence of their partners during maternal and child health follow-up.

Interviews were conducted in private rooms within the health facilities to ensure confidentiality and comfort, with interaction occurring exclusively between the researcher and each participant. Each interview lasted approximately 15–25 min, allowing sufficient time for participants to freely express their views without interruption.

The interview guide was pre-tested to assess the clarity, relevance, and appropriateness of the vocabulary and structure of the questions. A pilot interview was conducted to ensure that the instrument effectively captured the information necessary to address the study objectives, and minor adjustments were made accordingly.

The semi-structured interview guide covered several key domains, including: women's experiences attending maternal and child health services without their partners, perceived reasons for the absence of male partners during consultations, sociocultural expectations related to gender roles in maternal and child healthcare, economic or logistical barriers affecting partner participation; and women's perceptions of health services and strategies that could encourage greater male involvement (Supplementary Material S1).

These domains guided the interviews while allowing flexibility for participants to elaborate on issues they considered important.

The interviews were conducted by a trained researcher with experience in maternal and child health research. The researcher maintained a neutral and non-judgmental approach during the interviews to minimize potential influence on participants' responses. Awareness of the researcher's professional background and its potential impact on data interpretation was maintained throughout the data collection and analysis process. Regular reflection and discussion among the research team were conducted to ensure that interpretations remained grounded in participants' narratives.

#### Data analysis

Content analysis was conducted using an inductive qualitative approach, allowing themes to emerge directly from the data. All interviews were transcribed verbatim, and imported into MAXQDA for systematic analysis.

Two members of the research team independently reviewed and coded the transcripts. During the initial phase, open coding was used to identify meaningful segments of text related to women's perceptions of male partner involvement in maternal and child healthcare. Codes with similar meanings were grouped into broader categories and themes through an iterative analytical process.

To ensure analytical rigor, the two coders compared their coding results and discussed discrepancies during regular review meetings. Differences in interpretation were resolved through discussion until consensus was reached, resulting in a final coding framework agreed upon by the research team.

Data saturation was considered achieved when additional interviews did not generate new codes or thematic categories relevant to the research objectives. During the final stages of analysis, repeated patterns across participants' narratives indicated that the collected data were sufficient to capture the main dimensions of women's perceptions regarding male partner non-participation in maternal and child healthcare.

All sociodemographic data from the interviews with the women were digitized and exported to the Statistical Package for the Social Sciences (SPSS) version 28.0, to estimate the percentages of participants' sociodemographic variables, such as: age groups, marital status, place of residence, educational level, professional occupation, and religion.

### Ethical approval

Ethical approval for this study was obtained from the Gaza Institutional Bioethics Committee for Health (CIBS-Gaza) (Reference No. 129/CIBS-Gaza/2022, approved on June 9, 2022), as well as from the relevant institutional and provincial health authorities in Gaza Province. All participants were informed about the objectives and procedures of the study and provided voluntary informed consent prior to participation. For participants who were unable to read or write, consent was confirmed through an indelible ink fingerprint in the presence of a legal representative. Confidentiality and anonymity were ensured throughout the research process, and participants were informed of their right to withdraw from the study at any time without consequences.

## Results

This section presents the findings of the study based on women's narratives regarding the absence of male partners in maternal and child healthcare consultations. The results are organized into the following thematic categories: socio-demographic characteristics of participants, sociocultural and gender norms, economic constraints, fear of HIV testing, health system constraints, and women's perceptions of health services.

### Socio-demographic characteristics of study participants

Socio-demographic characteristics of 15 participants are presented in Table 1. Most participants were young, with 9/15 (60%) aged 18–24 years, 4/15 (26.7%) aged 25–29 years, and 2/15 (13.3%) aged 30–35 years. Regarding marital status, 7/15 (46.7%) participants were single, 1/15 (6.7%) was married, and 7/15 (46.7%) were in a common-law union. Most participants resided in urban areas 9/15 (60%), while 6/15 (40%) lived in rural areas.

**Table 1:** Socio-demographic characteristics of study participants.

Age, years	Number of participants	%
18–24	9	60
25–29	4	26.7
30–35	2	13.3
Marital status		
Single	7	46.7
Married	1	6.7
Common-law union	7	46.7
Residence		
Urban	9	60
Rural	6	40
Educational level		
Primary education	1	6.7
Secondary education	9	60
Higher education	5	33.3
Professional occupation		
Unemployed	4	26.7
Housekeeper	8	53.3
Informal trader	1	6.7
Public servant	2	13.3
Religion		
Christian	15	100
Other religions	0	0

In terms of educational attainment, 9/15 (60%) participants had completed secondary education, 5/15 (33.3%) had higher education, and 1/15 (6.7%) had only primary education. Professional occupations varied, with 8/15 (53.3%) participants working as housekeepers, 4/15 (26.7%) unemployed, 2/15 (13.3%) employed as public servants, and 1/15 (6.7%) engaged in informal trade.

All participants identified as Christian, 15/15 (100%), reflecting the religious composition of the study population. The participants were predominantly young and living in urban areas, with most having completed secondary education. Given the small qualitative sample, these characteristics should be interpreted only as descriptive of the participants included in the study rather than representative of the broader population.

### Sociocultural and gender norms

Participants frequently described sociocultural expectations that assign responsibility for maternal and child healthcare primarily to women. According to the women interviewed, pregnancy-related care, child vaccination, and routine health consultations are commonly perceived within the community as women's responsibilities. This perception contributes to the normalization of male absence from antenatal and child health consultations. Several participants explained that their partners believed attendance at health services was unnecessary or inappropriate for men, reinforcing traditional gender roles within the household.

“Healthcare is fundamental for well-being, but fathers do not always show concern for their children. Thus, it is left to women to carry this responsibility.” (P5).

“I have never come with him because he thinks it's unnecessary. He believes it's a woman's matter and doesn't like exposing personal life.” (P4).

“Sociocultural norms often place the responsibility for family planning, child vaccination, and maternal health care solely on women, highlighting the need to actively engage male partners in these aspects of family health.” (P13).

### Economic constraints

Although economic constraints and health system barriers are presented as separate themes, participants' narratives often indicated that these factors were interconnected. For example, limited financial resources and inflexible work schedules interacted with health facility operating hours, making it difficult for male partners to attend consultations.

“I know his presence is important because we are taking care of our child. However, he cannot come to the health facility due to lack of money for transport and the costs associated with the visit.” (P12)

“In my case, he does not refuse to come with me. On the contrary, he even enjoys it. But there are days he cannot attend because he is working.” (P5).

### Fear of HIV testing

Participants described fear of HIV testing as a sensitive factor influencing male partner attendance at maternal and child health services. Participants suggested that fear of HIV testing may discourage male partners from attending consultations.

“In South Africa, I went with him; we used to take the baby for weighing. Here at the hospitals, he has never come. He only comes when the child is sick. When we talk about HIV testing, he gets upset.” (P12).

In this narrative, this fear appeared linked to concerns about stigma, mistrust, and potential relationship conflict if HIV results were disclosed. Woman explained that discussions about HIV testing could create tension between partners, discouraging men from participating in routine maternal and child healthcare visits.

### Health system constraints

Participants also identified structural barriers within health facilities that discouraged male participation. These included limited consultation hours that conflicted with men's work schedules, long waiting times, and the perception that maternal and child health services were primarily designed for women.

“In my case, he does not refuse to come with me. On the contrary, he even enjoys it. However, there are days he cannot attend because the health facility operates during his working hours, making it difficult for him to accompany me.”(P5)

Some women also suggested that the physical environment and organization of services were not welcoming to male partners, reinforcing the perception that these services were intended primarily for women and children.

Despite these challenges, some women described strategies they used to encourage their partners' involvement. These included discussing the importance of child health, sharing information received during consultations, and inviting partners to attend appointments when possible. Although male participation remained limited, these narratives highlight women's active role in navigating social and institutional constraints to support family health.

### Satisfaction with health services

Participants expressed mixed perceptions regarding their experiences with health services at the Xai-Xai City Health Center. Several women reported satisfaction with the quality and efficiency of care.

“Yes, the conditions are good. The service is quick and efficient, and I always feel happy after being attended to.” (P10)

However, other participants highlighted concerns related to confidentiality and privacy during consultations.

“There is no privacy. Suppose they are testing me for HIV; that does not mean other nurses need to know my result.” (P13).

These contrasting perceptions suggest that while participants generally valued the availability and professionalism of health services, structural and organizational limitations particularly related to confidentiality may negatively affect patient experiences.

## Discussion

The findings of this study elucidate the complex interplay between sociocultural norms, economic pressures, HIV-related concerns, and health system barriers that shape male participation in Maternal and Child Health care in southern Mozambique. Rather than reflecting individual attitudes alone, these factors illustrate how gender expectations, economic structures, and institutional practices collectively influence men's engagement in reproductive health.

The sample was predominantly composed of young, urban women with secondary or higher education, reflecting a population group commonly accessing maternal and child health services in urban Mozambique. This pattern is consistent with national data showing that urban men in Mozambique tend to have higher levels of education and better access to health information and services (25–27).

This family structure is critical, as single women or those in informal unions often experience more limited partner support, as male partners may be physically absent or emotionally disengaged from pregnancy and childcare responsibilities (9, 28). Such absence is frequently reinforced by prevailing gender norms that define maternal and child health as predominantly female domains, discouraging men's participation in antenatal and postnatal care (9, 23, 24). Moreover, socioeconomic hardship and low educational attainment among male partners constrain their understanding of maternal health needs and reduce their capacity to engage meaningfully (29, 30).

The low male participation observed in Xai-Xai converges with trends observed across Sub-Saharan Africa. While in Xai-Xai male absence appears to be the norm, studies in similar contexts report participation rates of only 18% in Cameroon, less than 5% in Uganda, and approximately 15% in Kenya and Tanzania (9, 31). This regional consistency suggests that male absence is rooted in health systems that historically prioritize women as the primary entry point for MCH and Prevention of Mother-to-Child Transmission services (30, 32, 33).

Narratives from the participants confirm that pregnancy and child vaccination are socially constructed as exclusive responsibilities of women. This phenomenon can be interpreted through the lens of Hegemonic Masculinity, where the male role is strictly associated with economic provision and authority. Consequently, attending MCH consultations may be perceived as incompatible with socially valued masculine identities, leading to the symbolic construction of maternal care as a female domain (24, 34).

This account reflects gender norms widely reported across African contexts, where family care and reproductive health are predominantly constructed as women's responsibilities. Evidence from Mozambique shows that deeply rooted sociocultural norms and traditional gender roles shape patterns of communication about sexuality and health within families, often constraining male involvement (12, 25–27). In particular, discussions surrounding sexuality and reproductive health remain embedded in cultural taboos and social restrictions, which limit open dialogue and reinforce the marginal participation of certain family members, especially men (25).

The study suggests that interventions promoting interaction and communication among family members can help challenge prevailing gender norms and encourage behavioral change. In Mozambique, a study found that intergenerational dialogue sessions contributed to reducing taboos and improving communication about sexual and reproductive health within families. In this regard, promoting strategies that actively include men in discussions about family health may help transform social norms and foster shared responsibility for family well-being (25, 27).

Economic constraints and opportunity costs have been identified as significant barriers to male participation in maternal and child health services. Men often prioritize income-generating activities because time spent at health facilities represents a direct loss of earnings (35, 36). Studies also report that transportation costs and other indirect expenses, including long waits at clinics, deter men from attending maternal healthcare appointments (35, 36). Additionally, long distances and financial limitations associated with travel further reduce male partner involvement (37, 38). In contexts with substantial labor migration, such as work away from the home, prolonged physical absence during key gestational and pediatric follow-up periods further limits male engagement (3, 12, 28).

Fear of HIV testing and anticipated stigma have been identified as significant deterrents to the uptake of HIV testing in Sub-Saharan Africa and in Mozambique specifically. In rural communities of Mozambique, concerns about social stigma have been shown to cause individuals to avoid HIV testing, while interventions aimed at correcting misperceptions about stigma can increase uptake (39). The fear of HIV testing and concerns about disclosure of HIV status may discourage men from attending antenatal and maternal health services with their partners (3). At the population level, anticipated individual stigma is associated with reduced HIV testing among men, suggesting that expected negative social reactions contribute to avoidance of testing (39). Furthermore, systematic reviews across Africa report that fear of a positive result, anticipated partner or community reaction, and perceived lack of confidentiality in health facilities are major barriers to voluntary HIV testing (40–43).

Health system reforms may play a crucial role in addressing these barriers. In low-resource settings, feasible strategies could include extending clinic hours to accommodate working men, introducing couple-focused antenatal consultations, and providing dedicated waiting spaces for partners within maternal and child health services (25). Training healthcare providers to actively encourage male participation and improving confidentiality practices during HIV testing may also help reduce stigma and increase trust in health services. These health system barriers not only influence women's satisfaction but may indirectly discourage male participation by creating an unwelcoming environment for partners (34, 44).

The findings of this study are expected to contribute to strengthening the health system for the promotion of male health and the response to HIV. They may support multifaceted interventions, such as community-based education programs that challenge gender norms, health promotion campaigns targeting men, and the enhancement of HIV counseling and testing services tailored to their specific needs. Furthermore, these findings can facilitate access to health facilities through reforms that make services more male-friendly, significantly increasing male participation in healthcare. The implementation of these strategies, combined with public policies that promote gender equality and encourage male engagement, is essential to overcome fear-related barriers, expand HIV testing and treatment coverage, and improve health outcomes in high-prevalence populations, such as those observed in Gaza Province.

These findings have important implications for national maternal and HIV health strategies in Mozambique. Increasing male engagement could strengthen programs implemented by the Ministry of Health of Mozambique, particularly within initiatives aimed at preventing vertical transmission of HIV through the Prevention of Mother-to-Child Transmission Program. Policies that promote couple-based counseling, community education targeting men, and male-friendly service delivery models may help improve participation and ultimately contribute to better maternal and child health outcomes.

### Limitations of the study

An important limitation of this study is that it captures only women's perspectives on male participation. Women's narratives provide valuable insights into household dynamics and perceived barriers; however, they may also reflect interpretations or assumptions about men's motivations. In some cases, women may normalize male absence due to longstanding gender expectations or social pressures. Future research incorporating men's perspectives would provide a more comprehensive understanding of the relational and structural factors influencing male involvement in maternal and child health services.

The exclusion of men from the catchment area of the health facility resulted in findings that predominantly reflect the perceptions of women who access health services without their partners, which may have introduced bias. Although the study focused on understanding women's perceptions, the inclusion of male partners and healthcare professionals would have enabled a more comprehensive understanding of the phenomenon.

## Conclusions

Male involvement in maternal and child healthcare in Xai-Xai City remains limited due to the interaction of sociocultural norms, economic constraints, fear of HIV testing and health system barriers. Addressing these challenges requires integrated, context-sensitive of health system responses that move beyond individual-level interventions. To enhance male involvement in maternal and child healthcare, the health system interventions should focus on extending clinic hours to accommodate working men; introducing couple-focused antenatal consultations; strengthening confidentiality during HIV testing; implementing community-based interventions to challenge restrictive gender norms; and developing targeted health education campaigns for men. These strategies can support the integration of male-friendly services and promote shared responsibility in maternal and child healthcare, ultimately contributing to improved health outcomes.

## Data Availability

Publicly available datasets were analyzed in this study. This data can be found here: These data come from a thesis defended in 2022 by Maudy Simão Muchanga, in the Bachelor's degree program in Public Health at the University of São Tomás de Mozambique. Therefore, the group decided to use them for scientific publication.
