# Supplementary material for: Factors influencing male involvement in maternal and child health care from the health center of Xai-Xai city, Mozambique: a cross-sectional qualitative study
Source: Front Reprod Health. 2026 Apr 24;8:1746689. doi: 10.3389/frph.2026.1746689 (PMC13153094; doi:10.3389/frph.2026.1746689)
Supplement: Supplementary file 1 [file Datasheet1.pdf]

Apêndice III: Instrumento de recolha de dados

Guião de Entrevista

Versão 3.0, 26/05/2022

Querida irmã, peço a sua colaboração no sentido de melhorar a prestação dos cuidados de saúde da mulher e criança oferecidos pelo Centro de Saúde da Cidade de Xai-Xai para evitar problemas graves de saúde a mulher e criança, bem como ao seu parceiro. Por favor responde as perguntas que se seguem com calma, sem medo, pois as mesmas não são de carácter avaliativo. Garanto o anonimato e confidencialidade de toda informação. Antecipadamente agradeço a sua colaboração.

Nº de Entrevista: ___; Data: _____/____/ 2022; Local_________________.

1. Dados socio demográficos e económicos:

1.1. Dados Pessoais:

- Código: _______ Sexo: Feminino □, Residência________.

1.2. Proveniência

- Bairro 1 □; Bairro 8 □; Bairro 9 □; Bairro 11 □; Outros Bairros___________.

1.3. Ocupação:

- Estudante □; Doméstica □; Negociante □; Funcionário □.

1.4. Faixa etária:

- 18 -24 anos □; 25-29 anos □;30 -35 anos □.

1.5. Estado civil:

- Solteiro □; Casado □; Viúvo □; Separado / Divorciado □; União de facto □.

1.6. Raça

- Branca □; Negra □.

1.7. Religião

- Não professa nenhuma religião □; Cristã □; Islã □; outras__________.

1.8. Nível de escolaridade:

- Analfabeto □; Primário □; Secundário □; Ensino Profissional □; Superior □.

2. Percepção das participantes em relação aos cuidados de saúde da mulher e da criança.

2.1. Oque entende por cuidados de saúde?

____

____

____

____.

2.2. Qual é a sua percepção em relação aos cuidados de saúde da mulher e criança?

____

____

____

____.

2.3. Quais são os cuidados de saúde da mulher e criança prestados neste Centro de saúde?

____

____

____

____.

2.4. Qual é a importância dos cuidados de saúde da mulher e criança?

____

____

____

____.

3. Nível de percepção das participantes em relação a importância do acompanhamento dos seus parceiros a Unidades Sanitária.

3.1. Como tem sido o acompanhamento do seu parceiro durante as visitas a unidade sanitária para atendimento médico?

____

____

____

____.

3.2. Fala-me sobre a importância do acompanhamento do seu parceiro a unidade sanitária?

____

____

____

____.

3.3. De que forma o seu parceiro reage quando é convidado para visitar a unidade sanitária para atendimento médico?

____

____

____

____.

3.4. Com que frequência o seu parceiro tem visitado a unidade sanitária?

____

____

____.

3.5. Porque o seu parceiro não tem lhe acompanhado a unidade sanitária?

____

____

____

____.

3.6. Qual é o nível de confiabilidade do seu parceiro em relação aos cuidados de saúde oferecidos pela unidade sanitária?

____

____

____

____.

3.7. Que problemas de saúde que podem ser causados por não ir acompanhada com o seu parceiro a unidade sanitária?

____

____

____

____.

4. Nível de satisfação das mulheres em relação ao atendimento e cuidados de saúde prestados pelos profissionais de saúde.

4.1. Quais são as condições dos serviços prestados pelos profissionais de saúde nesta unidade sanitária?

____

____

____

____.

4.2. Como avalia o atendimento prestados pelos profissionais de saúde nesta unidade sanitária?

____

5. Atitudes dos profissionais de saúde em relação ao engajamento masculino

5.1. Quais são as medidas que os profissionais de saúde tomam em relação ao engajamento masculino?

5.2. Oque acha que os profissionais de saúde devem fazer para que haja maior participação dos parceiros das mulheres nos cuidados de saúde?

5.3. Que dificuldades o seu parceiro enfrenta para aderir aos cuidados de saúde a esta unidade sanitária?

Obrigado pela sua participação!
